# Occlusal vs non-occlusal modality of the loading protocol for oral implants in partially edentulous patients: a systematic review and meta-analysis

**DOI:** 10.1038/s41405-025-00347-3

**Published:** 2025-07-04

**Authors:** Panagiotis Kourkoutis, Rawand Shado, Ines Novo Pereira, David Madruga, Haidar Hassan

**Affiliations:** 1 Rey Juan Carlos University, Alcorcón, Madrid Spain; 2 Barts & The London School of Medicine & Dentistry, Queen Mary University, Institute of Dentistry, Royal London Dental Hospital, London, UK; 3 Faculty of Dental Medicine, University of Porto (FMDUP) Rua Dr. Manuel Pereira da Silva, Porto, Portugal; 4 Egas Moniz Center for Interdisciplinary Research (CiiEM), Egas Moniz School of Health & Science, Campus Universitário, Quinta da Granja, Caparica, Almada Portugal; 5 Barts & The London School of Medicine & Dentistry, Queen Mary University, Centre for Cutaneous Research, Blizard Institute of Cell and Molecular Science, London, UK

**Keywords:** Peri-implantitis, Dental implants, Occlusion, Fixed prosthodontics, Dentistry

## Abstract

**Background:**

Occlusal loading refers to a modality in which an implant-supported prosthesis is subjected to functional loading, maintaining contact with the opposing dentition from the onset of prosthetic placement. In contrast, non-occlusal loading represents a non-functional approach, wherein a provisional implant prosthesis is initially placed in infra-occlusion or fully relieved of contact with the opposing dentition, which is subsequently (at a later stage) followed by functional (occlusal) loading with the definitive prosthesis.

**Aim:**

To compare clinical outcomes in partially edentulous cases following an occlusal modality of loading versus non-occlusal modality of loading.

**Method:**

A search on Pubmed, Scopus and Embase databases was conducted to identify randomised controlled trials (RCTs) comparing occlusal versus non-occlusal modalities of implant loading in partially edentulous patients receiving implants with single crowns or fixed bridges, between January 1 (2004) to June 12 (2024), examining implant survival, complications and marginal bone loss (MBL) of implants. The inclusion criteria involved RCTs of evidence level II (Oxford Centre for Evidence-Based Medicine Levels of Evidence). For assessing bias in the included studies, the Cochrane Risk of Bias tool was used.

**Results:**

This review identified seven RCTs investigating 273 implants over 1–3 years follow-up periods. seven studies reported 1-year MBL data and three reported 3-year data. Publication bias was noted at the 1-year follow-up (*p* < 0.01) but not at 3 years (*p* > 0.05). Differences in MBL were not statistically significant at both 1 year (Hedges’ *d* = 0.01, *p* = 0.920, 95% CI: [−0.21, 0.24]) and 3 years (Hedges’ *d* = 0.01, *p* = 0.952, 95% CI: [−0.28, 0.30]). Differences in complication occurrences were not statistically significant (RR = 0.882, *p* = 0.759, 95% CI: [0.397, 1.964]). The nature of data on implant survival rates prevented a meaningful meta-analysis.

**Conclusion:**

For short-term periods of 1–3 years, no significant evidence supports clinical superiority in terms of complication rates and MBL between non-occlusal and occlusal modalities of implant loading. Future studies should explore functional and aesthetic aspects, as well as patient reported outcomes to determine any short-term differences or consider long-term follow-up with large sample sizes to detect significant clinical differences.

## Introduction

**Fig. 1:**
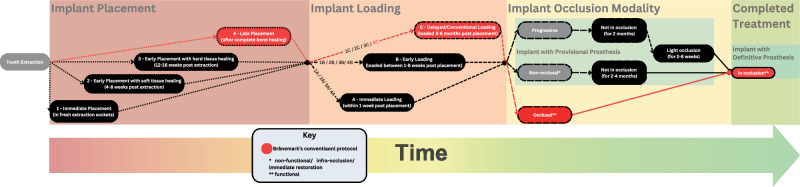
Clinical protocols for implant placement, loading and occlusal modality. Overview of implant treatment protocols categorised by timing of implant placement (immediate, early with soft tissue healing, early with hard tissue healing, late after complete bone healing), implant loading (immediate, early, delayed/conventional) and occlusal modalities (non-occlusal, progressive, non-occlusal). The framework highlights the integration of prosthetic phases and occlusal considerations in treatment planning.

Dental implant treatment has become an integral technique in dentistry to replace missing teeth. The main stages of the procedure include implant placement (surgically inserting the implant fixture into the jawbone), implant loading (attaching the prosthesis) and occlusion modality (staging the degree of occlusal contact in static and dynamic occlusion). There are many combinations of protocols for dental implant treatment to tackle different priorities (e.g., function and aesthetics, patient satisfaction, treatment longevity, duration and costs). The conventional technique, as described by Brånemark et al. [1], involves placing the implant at least 3 months after tooth extraction, to allow adequate bone healing and ensure tissue strength at the bone-implant interface. The final prosthesis is attached in occlusion 3-6 months after the implant placement. Over the years, studies have proposed variations to this conventional timeline. Figure 1 presents a summary of the timeline following a combination of different protocols to complete the implant treatment [1–5]. Among the variation of the protocols for implant loading, a recent scientometric analysis from 2021 revealed an increasing interest towards the immediate loading modality [6].

### Complete edentulism

A systematic review from 2021 showed that conventional/delayed loading was more favourable than immediate loading for removable prosthesis and non-splinted implants in edentulous mandible [7]. Conversely, a more recent study from 2024 concluded that there are no differences between conventional/delayed loading and immediate loading for implant-supported mandibular overdenture [8].

### Partial edentulism

Many systematic reviews have shown that different loading protocols do not significantly affect implant survival, complication rates or marginal bone loss (MBL) in partially edentulous cases [9–13]. Nevertheless, other systematic reviews reported differences between loading protocols, often favouring the conventional protocol [14–16]. In contrast, a cohort study from 2021 examining 10,871 implants over 22 years concluded that there is no significant difference between conventional/delayed loading and immediate loading [17].

### Anterior maxillary region and aesthetics

Recent systematic reviews provided significant insights into the outcomes of different implant loading protocols. For example, a study from 2023 demonstrated that the 1A protocol (Fig. 1) resulted in an acceptable survival rate and favourable aesthetic outcomes for single implants in the maxillary anterior region, with these outcomes maintained for up to five years [18]. Similarly, Pommer et al. found no significant differences between immediate and conventional/delayed loading protocols in maxillary single-tooth implants within the aesthetic zone [19]. Furthermore, Franscisco et al. reported no difference in aesthetic outcomes between immediate and early loading protocols for single implants [20].

### Rational behind this review

While recent systematic reviews have examined the effects of different loading protocols in fully edentulous arches, partial edentulism and the aesthetic zone, there is limited research on the different occlusion modalities within these protocols. Only one systematic review conducted a decade ago has evaluated the outcomes of occlusal versus non-occlusal modalities of loading [21]. Given the absence of contemporary evaluations of the body of evidence and an increasing interest in exploring the clinical effects of various implant loading protocols [6], we sought to provide an update on this topic and conduct a meta-analysis to compare occlusal versus non-occlusal temporary prostheses in partially edentulous cases.

## Methods

The PRISMA was followed for reporting this review (Supplement 1) [22, 23]. The PICO framework was used to structure the reporting of eligibility criteria [24]:

Population: Patients requiring dental implants aged greater than 18 years and medically healthy.

Intervention: Non-occlusal loading, defined as immediate, early or delayed loading the implant with a provisional prothesis out of occlusion within 2–4 months post implant placement, followed by a definitive prosthesis in occlusion (Fig. 1).

Comparison: Occlusal loading, defined as immediate, early or delayed loading the implant with a definitive prosthesis in occlusion in conjunction (Fig. 1).

Outcomes: Implant survival, complications and MBL.

### Search strategy

PubMed, Scopus and Embase were the main databases used for conducting the search for articles. The databases were searched from the 1^st^ of January (2004) to 12th of June (2024). Supplement 1a presents the key search terms used for articles retrieval.

### Study selection

The eligibility criteria (Supplement 1b) ensured that the selected studies focused on selecting randomised controlled trials conducted in hospital or clinical settings, involving adults aged 18–75, and comparing occlusal versus non-occlusal loading, on dental implant abutments, in partial edentulous cases. Studies had to report radiographic marginal bone loss, complications and survival rates. There were no restrictions on implant protocols, sample size or geographic location. Exclusions applied to studies without a comparative group, non-radiographic bone loss reporting, paediatric or special needs populations, restorative/endodontic treatments, non-dental procedures, animal studies, reviews, case reports, non-English articles and duplicate studies.

Two independent reviewers (PK and RS) carried out the study selection in a multi-stage process. First, they conducted an initial screening of titles and abstracts to identify studies that might meet the inclusion criteria. Next, they reviewed the full text of these potentially relevant studies. Any studies that did not satisfy the inclusion criteria were excluded. After the initial screening, the reviewers compared their selections. If they disagreed on a study’s eligibility and consensus could not be achieved, a third reviewer (HH) made the final decision.

### Data collection

In relation to each investigated study, data collection was completed independently by two reviewers (PK, RS). Another author (HH) reviewed extracted data and resolved any discrepancies.

Data extracted included: 1) Study Publication Details: authorship, year of publication, and country of origin. 2) Study Characteristics: demographic variables including total patients, sex and age. 3) Study Methodology: implant loading protocol, implant sites and follow-up. The selected studies were categorised using the Oxford Centre for Evidence-Based Medicine Levels of Evidence (OCEBM) classification system [25]. 4) Study Outcomes: implant survival rate, complications (fracture, loosening and periimplant mucositis) and marginal bone levels.

### Data preparation

When a particular data point was missing, we recorded this absence systematically as “Not Reported” (NR) in our analysis. For continuous variables, such as age, if separate values were provided for each group and no statistically significant difference was found between them, we combined the mean and standard deviation values for presentation in our table.

### Risk of bias

The Risk of Bias 2 (RoB2) assessment tool was used to assess risk of bias for RCTs (Level II) [26]. For each study, the overall bias was given based on the highest bias score for each decision category. For example, if the highest score of ‘High’ was estimated for one or more decision categories, then the overall bias was considered ‘High.

### Data analysis, statistical methods and data visualisation

In this meta-analysis, a random-effects model was employed, and the standardised mean difference (SMD) was computed using Hedge’s *d*, with a focus on confidence interval overlap with the no-effect threshold (SMD = 0) and effect size thresholds as per Cohen’s classification [27]: small (0.2), moderate (0.5) and large (0.8). An effect was considered statistically significant if the 95% confidence interval exceeded the moderate (0.5) threshold.

Publication bias was assessed through visual examination of funnel plot asymmetry and cumulative meta-analysis plots, supplemented by Egger’s Test. To evaluate heterogeneity, the statistical I² test and the Cochrane Q test were applied, along with visual inspection of confidence intervals. Heterogeneity levels were classified based on Higgins and Thompson’s thresholds [28]: low (25%), medium (50%), and high (75%).

Statistical analyses and data visualisation were conducted using Python programming language, employing libraries such as Pandas, NumPy, Statsmodels, SciPy and Plotly.

## Results

### Studies included

**Fig. 2:**
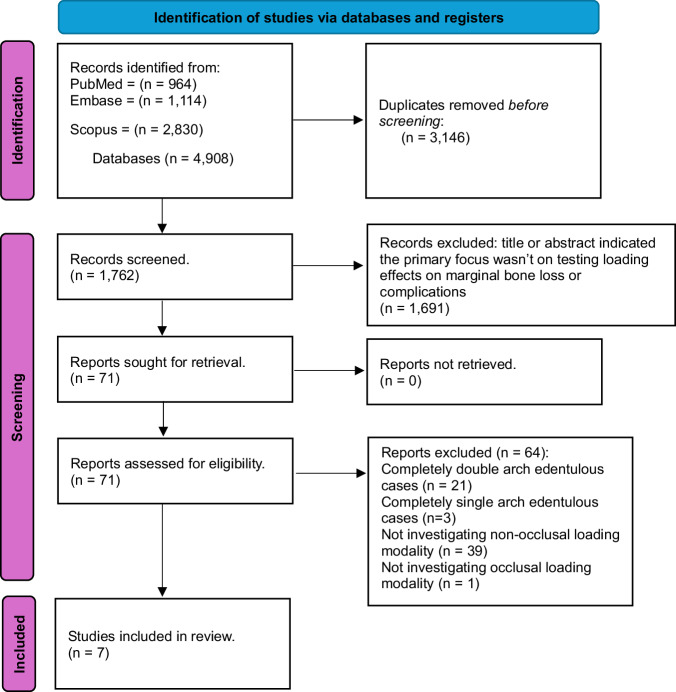
PRISMA flowchart. A flowchart demonstrating the identification, screening and the inclusion process of the included articles in this review.

Figure 2 shows the PRISMA flowchart representing study selection and inclusion [22, 23]. The initial search resulted in 4908 papers for all databases combined. This was trimmed down to 1762 after duplicates were removed. Following the first-stage screening of titles and abstracts, 71 articles (considered potentially suitable by at least one reviewer) qualified for full-text screening. After full-text reading, seven studies, with 273 implants in total and 1–3 years follow-up periods, met the inclusion criteria, and 64 papers were excluded (see Fig. 2 for reasons for exclusion). Table 1 reports the studies and their characteristics, which included seven RCTs [5, 29–34]. The studies primarily focused on immediate loading [29–31, 33, 34], with one study on early loading [32], and one on delayed/conventional loading [5].

**Table 1:** Characteristics of the included studies.

First author, year, country	Study design (level of evidence - risk of bias)	Population	Groups	Implant site	Implant prosthesis span	Follow-up (months)
Alassah et al., 2022, Syria	Double blind splitmouth randomised controlled trial (II-low)	Implants: 24 Males: 2 Females: 7 Age: 51 ± 12.6	Implants: 12 Protocol: Early non-occlusal loading vs Implants: 12 Protocol: Early occlusal loading	Mandibular Posterior	NR	12
Vogl et al., 2019, Austria	Single blind randomised controlled trial (II-low)	Implants: 19 Males: 7 Females: 12 Age: 54 ± 11.9 (33–70)	Implants: 11 Protocol: Immediate non-occlusal loading vs Implants: 8 Protocol: Immediate occlusal loading	Mandibular Posterior	Combination (both single crowns and short bridges)	36
Esposito et al., 2018, Italy	Single blind randomised controlled trial (II- moderate)	Implants: 40 Males: 19 Females: 21 Age: 43.4 (18–65)	Implants: 20 Protocol: Immediate non-occlusal loading vs Implants: 20 Protocol: Immediate occlusal loading	Mandibular & Maxillary Posterior	Single-unit crown	36
Degidi et al., 2010, Italy	Single blind randomised controlled trial (II-moderate)	Implants: 100 Males: NR Females: NR Age: 45.1 ± 9.1	Implants: 50 Protocol: Immediate non-occlusal loading vs Implants: 50 Protocol: Immediate occlusal loading	Mandibular Posterior	Single-unit crown	36
Cannizzaro et al., 2010, Italy	Multi-centre single blind randomised controlled trial (II-low)	Implants: 35 Males: 17 Females: 23 Age: 38.5 (18–55)	Implants: 18 Protocol: Immediate non-occlusal loading vs Implants: 17 Protocol: Immediate occlusal loading	Mandible & Maxilla	Single-unit crown	12
Degidi et al., 2009, Italy	Single blind randomised controlled trial (II-low)	Implants: 60 Males: 27 Females: 33 Age: 31.5 ± 11.8 (18–55)	Implants: 30 Protocol: Immediate non-occlusal loading vs Implants: 30 Protocol: Immediate occlusal loading	Maxillary Anterior	Multi-unit bridge	36
Lindeboom et al., 2006, Netherlands	Randomised controlled trial (II-moderate)	Implants: 50 Males: 17 Females: 31 Age: 42.3 ± 13.1 (19–78)	Implants: 25 Protocol: Immediate non-occlusal loading vs Implants: 25 Protocol: Immediate occlusal loading	Maxillary Anterior	Single-unit crown	12
Appleton et al., 2004, Taiwan	Randomised controlled trial (II-high)	Implants: 20 Males: NR Females: NR Age: NR	Implants: 10 Protocol: Delayed progressive (non-occlusal loading) vs Implants: 10 Protocol: Delayed occlusal loading	Maxillary Posterior	Single-unit crown	12

A table showing a summary of the characteristics of each included study in this review.

*NR* Not reported

### Sample size and statistical power

The seven trials reviewed employed different strategies to justify their sample size calculations, with direct consequences for statistical power and interpretability. Degidi et al. based their calculation on expected differences in early implant failure (3.7% vs 1.5%) [30], determining that 46 subjects were required and recruiting 50 implants split evenly between two groups; the study therefore met its own power target for the primary outcome of survival. Alassah et al. used pilot data and G*Power to show that 18 implants would provide 80% power in a split-mouth design [32]; they increased the sample to 24 implants, so the experiment was adequately powered for large effects despite its small absolute size. Lindeboom et al. performed an a-priori equivalence calculation, indicating that 21 implants per arm were needed to detect a 10-point ISQ difference [34]; they enroled 25 per arm, achieving the desired power for stability but not for rarer events such as biological failure.

In contrast, several studies were under-recruited relative to their stated goals. Esposito et al. calculated that 154 patients per group would yield 90% power for implant failure [31], yet enrolled only 40 in total, rendering the trial grossly under-powered for its primary endpoint. Cannizzaro et al. likewise planned for 154 patients per arm [29], but managed to recruit just 20 per arm across four centres, a shortfall acknowledged by the authors and reflected in wide confidence intervals around survival estimates. Two pilot investigations, Vogl et al. (20 patients, 52 implants) and Appleton et al. (20 subjects, 23 implants) [5, 33], did not attempt formal power calculations; both explicitly framed their trials as exploratory, signalling that any non-significant findings must be interpreted as inconclusive rather than demonstrative of equivalence.

Collectively, only three of the seven trials met their own power requirements, and even those were sized to detect relatively large differences in early failure or ISQ rather than the modest variations in MBL that dominated contemporary clinical interest.

### Outcome measures and confounding variables

All seven randomised trials relied on broadly similar primary outcomes namely, implant or prosthesis failure, peri-implant marginal bone change and indices of mechanical stability. Esposito et al. recorded prosthesis failure, implant mobility, biological and prosthetic complications, serial marginal bone levels and a pink-aesthetic score assessed from calibrated photographs, supplementing these with patient-reported chair-time and satisfaction [31]. Vogl et al. followed a comparable core set but added Periotest resonance values at baseline, 12 and 36 months and a visual-analogue questionnaire covering aesthetics, phonetics, hygiene access and chewing comfort [33]. Degidi et al. extended the follow-up to three years, collecting ISQ, soft-tissue probing depths and gingival margin levels alongside radiographic bone loss and early adverse events [30].

Alassah et al. measured insertion torque, longitudinal ISQ at 0–12 weeks and bone loss at 12 weeks and one year, declaring 100% survival as a binary endpoint [32]. Appleton et al. relied on serial digital subtraction radiography to quantify crestal height change and nine-zone bone-density gradients over 12 months, without formal failure or aesthetic indices [5]. Cannizzaro et al. working with zirconia one-piece fixtures, tracked early failure, complications and 1-year bone loss, the latter measured on calibrated parallel radiographs by a blinded assessor [29]. Lindeboom et al. specified ISQ at six months as the primary endpoint, with 12-month bone loss, gingival-papilla scores and survival as secondary measures [34].

Interpretation of those outcomes is conditioned by a collection of potential confounders that varied by protocol. Mechanical factors dominated: every study imposed a minimum insertion-torque threshold (≥25 N cm [30] or ≥35 N cm [29, 31, 32]), yet Esposito et al. reported three fixtures loaded below target [31], and Cannizzaro still observed a 12.5% early failure rate despite universal high torque [29].

Surgical and anatomical heterogeneity added further nuance. Cannizzaro included immediate post-extraction cases (40% of which failed) alongside healed sites [29], whereas the other trials excluded sockets or posterior maxillary bone of poor quality, producing different baseline risk profiles. Appleton restricted recruitment to single premolars in the posterior maxilla to standardise bone density [5], while Vogl confined implants to the posterior mandible [33], yet allowed diverse opposing dentitions that could alter functional load.

Patient-level variables were unevenly distributed. Alassah’s twelve-subject split-mouth study suffered a pronounced gender imbalance [32], whereas Cannizzaro stratified smokers [29], but randomised a bruxist who experienced early failure, highlighting parafunction as an unmeasured confounder.

Finally, operator and centre effects complicated multicentre designs: Cannizzaro’s four active centres differed in failure and complication counts despite identical protocols [29], and Esposito documented deviations in implant length and torque at individual sites [31]. Such clustering inflates variance and can mask true differences between functional and non-functional loading strategies.

### Risk of bias

**Fig. 3:**
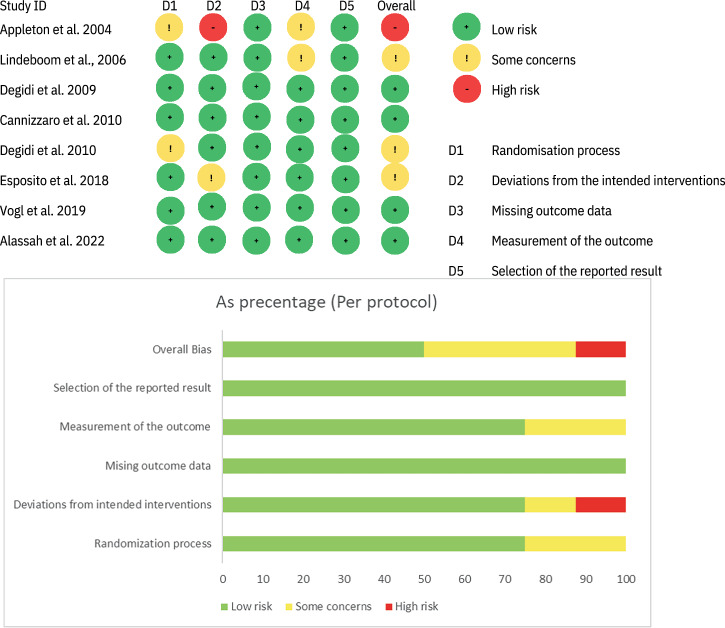
Risk of bias and level of evidence. A diagram showing the bias level within studies.

The risk of bias was assessed as low in three studies [29, 32, 33], with some concerns acknowledged in three studies [30, 31, 34] (Fig. 3). The other RCT was judged at high risk of bias due to unreported baseline characteristics of the test and control groups [5], as well as insufficient details regarding non-protocol-specific management of patients.

### Survival

Most of the studies reported a 100% survival rate in at least one group, while one study did not report survival rates at all [5]. The nature of this data creates a significant challenge for conducting a meaningful meta-analysis on survival rates, especially considering the relatively small sample sizes in the relatively short follow-up periods (1–3 years). These sizes are inadequate for detecting small differences in implant survival rates over the short term. From a statistical perspective, limited follow-up periods hinder the observation of long-term failure events, which are often required for accurately estimating survival probabilities. Furthermore, the high rate of censored data (implants surviving without failure during the study period) reduces the observed variance and increases the likelihood of Type II errors, making it challenging to detect meaningful differences. This restriction in variance may also inflate effect size estimates or bias the meta-analytic model, especially when survival rates are dichotomous and tend toward the upper boundary (100%).

### Complications

One study did not report on complication occurrences [5], while another reported no complications in either group after a one-year follow-up [32]. Five studies documented complications for both groups after one year of follow-up [29–31, 33, 34]. A meta-analysis of these results revealed no statistically significant difference in complication rates between occlusal and non-occlusal implant loading (RR = 0.882, *p* = 0.759, 95% CI: [0.397, 1.964]). The analysis also indicated minimal heterogeneity (I² < 0.01%, *p* = 0.484), suggesting consistency across the included studies.

### Marginal bone loss

**Fig. 4:**
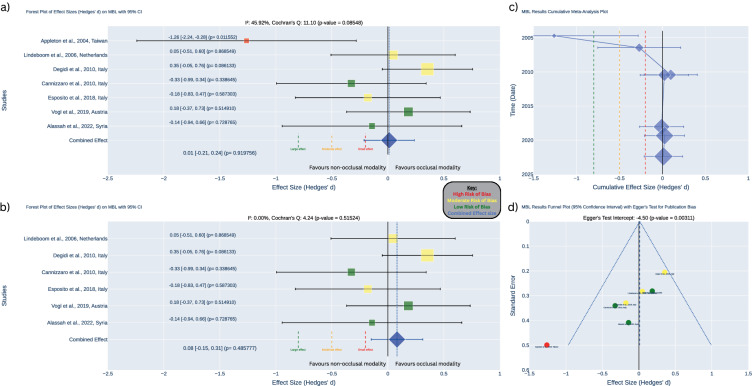
1-year meta-analysis of marginal bone loss (MBL). **a** Forest plot illustrating effect sizes (Hedges’ *d*) for MBL with 95% confidence intervals across studies (without the high risk of bias study). **b** Forest plot illustrating effect sizes (Hedges’ *d*) for MBL with 95% confidence intervals across studies (with the high risk of bias study). **c** Cumulative meta-analysis plot tracking changes in the combined effect size estimates over time. **d** Funnel plot assessing publication bias with Egger’s test.

After one year of follow-up, the meta-analysis of the eight studies showed no statistically significant difference in MBL between occlusal and non-occlusal loading of implants at 1–3 years. This was true both when including the high bias study (Hedges’ *d* = 0.01, *p* = 0.920, 95% CI: [−0.21, 0.24]) [5], and when the study was excluded (Hedges’ *d* = 0.08, *p* = 0.486, 95% CI: [−0.15, 0.31]). In both cases, the confidence intervals did not fall within the range of a meaningful effect size. The included studies showed low heterogeneity (I² = 45.92%, *p* = 0.085) and there was significant evidence of publication bias, as indicated by funnel plot asymmetry and Egger’s test (intercept = −4.50, *p* = 0.003) (see Fig. 4). In addition, the cumulative meta-analysis plot revealed a chronological trend of publication bias. Studies between 2005 and 2010 increasingly shifted the cumulative effect size toward the threshold for benefit (see Fig. 4).

**Fig. 5:**
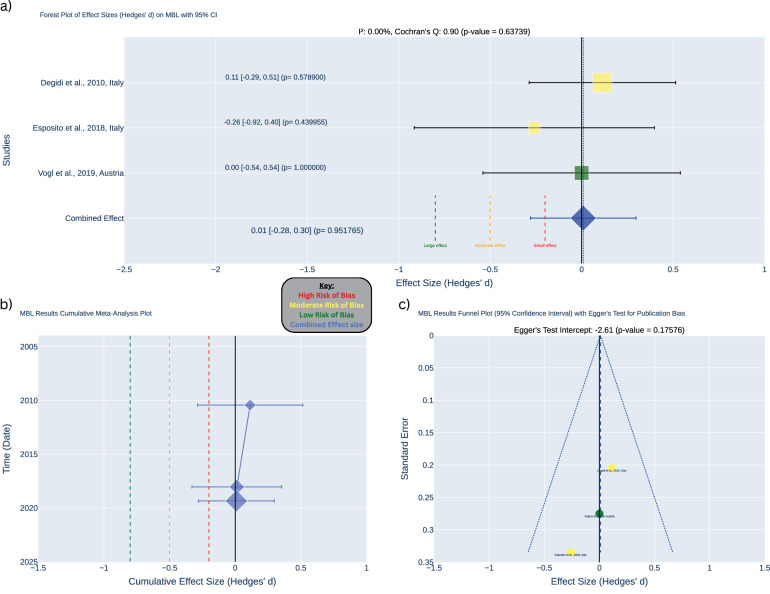
3-year meta-analysis of marginal bone loss (MBL). **a** Forest plot illustrating effect sizes (Hedges’ d) for MBL with 95% confidence intervals across studies. **b** Cumulative meta-analysis plot tracking changes in the combined effect size estimates over time. **c** Funnel plot assessing publication bias with Egger’s test.

After three years of follow-up, the meta-analysis of the three studies [30, 31, 33] showed no statistically significant difference in MBL between occlusal and non-occlusal loading of implants (Hedges’ *d* = 0.01, *p* = 0.952, 95% CI: [−0.28, 0.30]). The confidence intervals did not fall within the range of a meaningful effect size. The included studies showed low heterogeneity (I² < 0.01%, *p* = 0.637). No significant publication bias was detected, as evidenced by the symmetrical funnel plot and Egger’s test (intercept = −2.61, *p* = 0.176) (see Fig. 5).

## Discussion

The findings of this review suggest that, at a follow-up period of 1–3 years, there is no significant difference between occlusal and non-occlusal loading modalities in terms of complication rates and MBL outcomes.

Among the seven trials investigating 273 implants, only one study demonstrated a statistically significant reduction in MBL favouring the non-occlusal loading modality [5]. Data presented by three additional studies showed a non-significant trend favouring the non-occlusal modality [29, 31, 32], while another three studies reported a non-significant preference for the occlusal modality [30, 33, 34]. The pooled effect size revealed no statistically significant difference between the two occlusal modalities with no meaningful effect in terms of MBL.

Although we need to exercise caution in interpreting these findings because of the small number of studies, these findings appear to be largely in line with the previous meta-analysis by Chrcanovic (2014) [21], which reported a RR of 0.87 (95% CI: 0.44–1.75, *P* = 0.70), closely comparable to our result. Moreover, we found that the exclusion of the study with high risk of bias did not significantly affect the results (*p* > 0.05) [5]. Regardless of whether this study was included or not, it still resulted in a non-significant overall difference in MBL (*p* > 0.05). In addition, the included studies were often limited by publication bias, suggesting that the results may be overestimated. Nonetheless, the differences in clinical outcomes between the groups remained non-significant. This indicates that despite the potential publication bias, the clinical outcomes were not significantly different, demonstrating the reliability of the findings. It is, however, important to bear in mind that the published studies increasingly shifted the cumulative effect size toward the threshold for benefit, suggesting a tendency to report results that minimised potential negative outcomes or increased the perception of positive effects over time.

### Results against existing literature

In comparison to other systematic reviews from the last decade, there is a consensus: occlusal contact at the time of implant loading does not adversely affect outcomes in the short term. Our results align closely with the meta-analyses by Chrcanovic et al. and Sommer et al. [14, 21], both found no significant difference in MBL between immediate functional vs immediate non-functional loading. In fact, Sommer’s data suggested marginally less bone loss with immediate (especially non-occlusal) loading than with delayed loading, reinforcing our observation that an initial period of relieved occlusion offers no clear bone-healing advantage over full occlusal loading. This was further supported by Chen et al. [35, 36], that noted comparable bone levels and peri-implant health whether or not implants were immediately loaded and regardless of the occlusal scheme. Where minor differences were noted (e.g. Sanz-Sánchez et al. reported a slightly higher early failure risk with occlusal loading), the absolute survival rates remained high in both groups, and these differences did not translate into any long-term marginal bone disparities or complication trends. Crucially, none of the systematic reviews found any increase in biological complications (such as peri-implantitis or sustained peri-implant mucosal inflammation) or prosthetic complications attributable to occlusal vs. non-occlusal loading protocols in the evaluated timeframe.

In summary, the body of evidence from 2014 to 2024 concurs with our systematic review: for partially edentulous patients, implants loaded in occlusion achieved comparable short-term MBL and complication rates as implants loaded initially in a non-occluding fashion followed by occlusion loading. These findings suggest that clinicians can choose an occlusal or non-occlusal immediate loading approach based on practical considerations (prosthesis type, patient comfort, etc.) rather than fear of jeopardising bone integration. The lack of significant differences over 1–3 years indicates that occlusal loading does not intrinsically cause MBL or implant failure in the context of proper case selection and implant stability. This consensus across systematic reviews validates the current review’s conclusion that neither loading modality holds a clear biologic or mechanical advantage in the early years of function. Any slight differences in outcome (e.g. a non-significant ~0.1 mm bone difference) are clinically negligible. That said, the present review’s findings are well supported by the broader literature: occlusal and non-occlusal loading protocols are equally viable for partial edentulism in terms of maintaining crestal bone and avoiding complications in the short term.

### Study limitations and future research

Across all studies, small sample sizes, deviations from protocols and incomplete outcome measures (e.g., patient-reported outcomes) were notable limitations, which may have influenced the reliability of the findings. For instance, some studies reported a small sample size that limited the statistical power for detecting low-incidence outcomes or complications and/or the certainty of findings [33–35]. Furthermore, the lack of stratified results by key confounders (e.g. smoking, periodontal disease) also raises concerns about the robustness of the findings. Noteworthy that the lack of studies assessing patient-reported outcomes (e.g. pain, quality of life and patient satisfaction), in addition to the traditional clinical outcomes (e.g., implant survival and stability, bone level changes and gingival health), may have prevented a holistic understanding of the clinical impact of the interventions.

We emphasise the need for additional high-quality RCTs to strengthen the evidence base. Future research should aim for larger sample sizes to increase the statistical power and allow for subgroup analyses. Additionally, a more comprehensive assessment of clinical outcomes considering the patient perspectives, soft tissue health and functional outcomes, could provide a better understanding of the treatment success. Controlling for potential confounders like smoking, oral hygiene practices and patient demographics (e.g., age, gender) could also improve the general applicability of future studies. In addition, blinding of examiners and statisticians may ensure objectivity in assessments.

### Implications for clinical practice and future trials

It is still important to regularly monitor dental implants and the associated MBL. Currently, there is no significant evidence in the literature to support strong clinical recommendations regarding specific implant occlusion modalities.

At the clinical level, there is no conclusive evidence to suggest that one modality is superior to another. Future trials should focus on functional, aesthetic and patient-reported outcomes for short-term studies (1–5 years) or conduct long-term studies (10–30 years) on clinical outcomes.

Nevertheless, from a bone remodelling and physiological perspective, we contend that studies extending beyond 3 to 5 years may offer limited additional value to the existing body of literature.

In all non-occlusal loading modalities, even under the longest duration, the prosthesis remains unloaded for a maximum duration of 4 months. Subsequently, these modalities transition to full loading, analogous to fully conventionally occlusally loaded groups. Any substantial alterations in bone levels attributable to non-occlusal loading modalities are likely to manifest during the initial months, extending up to 1–2 years following the placement of the definitive prosthesis and the commencement of loading.

We suggest that once non-occlusally loaded implant are subjected to occlusal loading, their clinical performance aligns closely with that of occlusally loaded implants after an initial period of full loading. However, the precise duration of this period remains indeterminate. Consequently, we propose that any significant differences in MBL, failure rates, complication prevalence and survival outcomes between the two groups are most likely to emerge within the first 2 years following the establishment of full loading in both implant groups. Notwithstanding, this hypothesis necessitates validation through a well-designed clinical study.

Given the high standard of implant survival rates over the long term, very large sample sizes and extended follow-up periods are necessary to detect statistically significant differences in survival. Therefore, it is more practical to concentrate on monitoring MBL and complication rates over time with reasonably sized samples.

### Limitations of this review

As outlined in the search strategy, we limited publications to those in English, which may have led to the exclusion of some relevant evidence.

The eligibility criteria for this study were restrictive, focusing only on comparative studies. Including non-controlled studies, such as case series, might have provided additional insights that could confirm or alter the conclusions of this study.

All clinical studies included in this review had short term follow-up (1–3 years), limiting scope for long term differences between occlusal and non-occlusal modalities. Finally, the present review was not previously registered. This increases the risk of an unplanned duplication and does not allow to confirm that the meta-analysis methods were carried out as planned.

## Conclusion

This review demonstrates that, in partially edentulous patients, implants restored either in full functional occlusion or initially relieved of occlusal contact (2–4 months) perform equivalently during the first three years. Pooled analyses revealed negligible, statistically non-significant differences in marginal bone loss at both one year (Hedges’ *d* ≈ 0.01; 95% CI −0.21 to 0.24) and three years (Hedges’ *d* ≈ 0.01; 95% CI −0.28 to 0.30). Biological and prosthetic complication rates were likewise indistinguishable between modalities (pooled risk ratio ≈ 0.882; 95% CI 0.397–1.964) and implant survival exceeded 97% in nearly every trial, precluding a meaningful meta-analysis of failure. Individual studies showed results scattered around the null: four favoured occlusal loading [5, 29, 31, 32], and three favoured non-occlusal loading [30, 33, 34], yet none produced a significant effect after accounting for sampling error. Although funnel-plot asymmetry suggests some publication bias at one year and many trials were small with limited follow-up, the overall body of evidence points toward clinical equivalence in the short term.

From a clinical perspective, these findings allow the choice of either occlusal or infra-occlusal provisionalisation to be governed primarily by prosthetic logistics, aesthetic demands and patient comfort rather than by concerns about negatively affecting crestal bone or increasing early complications, provided that sound primary stability and favourable occlusal risk factors are present.

Nevertheless, the certainty of evidence is tempered by short observation windows, small cohort sizes and selective reporting. Robust confirmation will require trials with larger, well-stratified samples, protocol pre-registration, consistent reporting of negative findings, longer follow-up beyond five years and the routine inclusion of patient-reported outcome measures to capture functional and aesthetic perceptions that may emerge after occlusion is established. Until such data become available, the current literature supports the view that neither occlusal nor non-occlusal loading offers a clinically relevant advantage during the first three years after implant placement in partially edentulous patients.

## Supplementary information

PRISMA 2020 Checklist

Supplement 1a 1b

## Data Availability

The data that support the findings of this study are available from the corresponding author upon reasonable request.
