# Supplementary material for: Occlusal vs non-occlusal modality of the loading protocol for oral implants in partially edentulous patients: a systematic review and meta-analysis
Source: BDJ Open. 2025 Jul 4;11:63. doi: 10.1038/s41405-025-00347-3 (PMC12227773; doi:10.1038/s41405-025-00347-3)
Supplement: Supplementary file 2 — Supplement 1a 1b [file 41405_2025_347_MOESM2_ESM.docx]

**Supplement 1a**. Search key words.

A table showing the search strategy employed in PubMed, Scopus, Embase and Google Scholar to identify the articles for this review.

| **PubMed search** | **Scopus** | **Embase** |
| --- | --- | --- |
| ("implant*"[All Fields] AND "occlu*"[All Fields]) AND ((clinicaltrial[Filter]) AND (2004:2024[pdat])) | TITLE-ABS-KEY ( ( implant* ) AND ( occlu* ) ) AND ( LIMIT-TO ( SUBJAREA , "DENT" ) ) AND ( LIMIT-TO ( EXACTKEYWORD , "Human" ) OR LIMIT-TO ( EXACTKEYWORD , "Humans" ) OR LIMIT-TO ( EXACTKEYWORD , "Dental Implants" ) OR LIMIT-TO ( EXACTKEYWORD , "Dental Prosthesis, Implant-Supported" ) OR LIMIT-TO ( EXACTKEYWORD , "Adult" ) OR LIMIT-TO ( EXACTKEYWORD , "Tooth Occlusion" ) OR LIMIT-TO ( EXACTKEYWORD , "Dental Prosthesis Design" ) OR LIMIT-TO ( EXACTKEYWORD , "Dental Restoration Failure" ) OR LIMIT-TO ( EXACTKEYWORD , "Dental Abutments" ) OR LIMIT-TO ( EXACTKEYWORD , "Tooth Crown" ) ) | (implant* AND occlu* AND ([controlled clinical trial]/lim OR [randomized controlled trial]/lim) AND [english]/lim AND [clinical study]/lim AND ([embase]/lim OR [medline]/lim OR [embase classic]/lim OR [pubmed-not-medline]/lim) AND [2004-2024]/py |

**Supplement 1b**. Inclusion and exclusion criteria.

A table showing the inclusion and exclusion criteria used during the articles selection process which led to the final inclusion of the studies in this review.

| **Inclusion criteria** | **Exclusion criteria** |
| --- | --- |
| - Hospital or clinical setting - Adult population (18-75 years old) - Randomized controlled trials - Studies employing occlusal vs non-occlusal loading modalities on dental implant abutment - Studies reporting radiographic marginal bone loss, complications or survival. - The investigation period is between 01st of January 2004 and 12th of June 2024 - No restrictions on implant placement protocol - No restrictions on implant load protocol - No restriction on study sample size - No restrictions on geographic location | - Studies without a comparative group - Studies not reporting radiographic bone loss - Paediatric or special needs population - Restorative or endodontic treatment - Non-dental procedures - Animal studies, books, reviews, case reports, editorials, letters, commentaries, or conference abstracts - Duplicate studies - Non-English articles |
